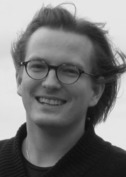# More than Proton Detection—New Avenues for NMR Spectroscopy of RNA

**DOI:** 10.1002/chem.201903355

**Published:** 2019-10-22

**Authors:** Robbin Schnieders, Sara Keyhani, Harald Schwalbe, Boris Fürtig

**Affiliations:** ^1^ Institute for Organic Chemistry and Chemical Biology Center for Biomolecular Magnetic Resonance (BMRZ) Johann Wolfgang Goethe-Universität Frankfurt Max-von-Laue-Str. 7 60438 Frankfurt Germany

**Keywords:** carbon direct detection, heteronuclear detection, nitrogen direct detection, NMR, RNA

## Abstract

Ribonucleic acid oligonucleotides (RNAs) play pivotal roles in cellular function (riboswitches), chemical biology applications (SELEX‐derived aptamers), cell biology and biomedical applications (transcriptomics). Furthermore, a growing number of RNA forms (long non‐coding RNAs, circular RNAs) but also RNA modifications are identified, showing the ever increasing functional diversity of RNAs. To describe and understand this functional diversity, structural studies of RNA are increasingly important. However, they are often more challenging than protein structural studies as RNAs are substantially more dynamic and their function is often linked to their structural transitions between alternative conformations. NMR is a prime technique to characterize these structural dynamics with atomic resolution. To extend the NMR size limitation and to characterize large RNAs and their complexes above 200 nucleotides, new NMR techniques have been developed. This Minireview reports on the development of NMR methods that utilize detection on low‐γ nuclei (heteronuclei like ^13^C or ^15^N with lower gyromagnetic ratio than ^1^H) to obtain unique structural and dynamic information for large RNA molecules in solution. Experiments involve through‐bond correlations of nucleobases and the phosphodiester backbone of RNA for chemical shift assignment and make information on hydrogen bonding uniquely accessible. Previously unobservable NMR resonances of amino groups in RNA nucleobases are now detected in experiments involving conformational exchange‐resistant double‐quantum ^1^H coherences, detected by ^13^C NMR spectroscopy. Furthermore, ^13^C and ^15^N chemical shifts provide valuable information on conformations. All the covered aspects point to the advantages of low‐γ nuclei detection experiments in RNA.

## Graphical abstract

**Enlightening the blind spots**: Newly established carbon‐ and nitrogen‐detection NMR experiments allow characterization of RNA structure and dynamics where traditional proton‐based techniques fail (see figure).

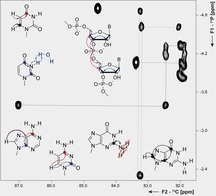

## 1 Introduction

Since the development of multidimensional NMR spectroscopy and the availability of isotope‐labeled RNAs, NMR spectroscopy has contributed more than 40 % of all RNA structures in databases. Considerable challenges, however, remain for the structure determination particularly of large RNAs and their complexes by biomolecular NMR spectroscopy. Often, the maximum concentration, at which RNA and RNA–protein complexes can be prepared for NMR studies, does not exceed 50 μm, either for solubility reasons or for availability of sample, and thus all NMR experiments have to be optimized to maximize the signal‐to‐noise ratio. The multitude of current approaches to achieve maximal signal‐to‐noise ratio have been recently summarized.^1^

Multidimensional NMR pulse sequences that rely on excitation and detection of the most sensitive nuclei protons (where ‘proton“ refers to ^1^H) have for long been the experimental gold standard in the field of biomolecular NMR spectroscopy. Since 1957,^2^ a plethora of studies have unravelled structure and dynamics of proteins and nucleic acids. Especially for the characterization of RNAs, NMR has led to new fundamental insight including transient base‐pair states modulating replication and transcription,^3^ long‐lived *meta*‐stable secondary structures controlling gene‐regulation during transcription^4^ and translation,^5^ and the structure of a viral packaging signal,^6^ to name only a few. The advantages of proton‐detected experiments apparently stem from the high natural abundance of the ^1^H‐isotope and its highest sensitivity of all NMR‐active and stable nuclei.

However, also proton‐detected experiments exhibit difficulties for NMR studies of RNAs. Chemical‐shift dispersion is limited for all their resonances with the exception of imino protons. Further, the proton‐density within the nucleobases of RNA is low and many nucleobase‐protons are susceptible to conformational and solvent exchange. Thus, methods to circumvent these problems are needed and we here summarize recent developments in heteronuclear‐detected experiments (^13^C, ^15^N, ^19^F, and others). In combination with the traditional proton‐detected experiments, they will open new possibilities in the structural description of RNA dynamics and function at atomic resolution.

**Figure 1:**
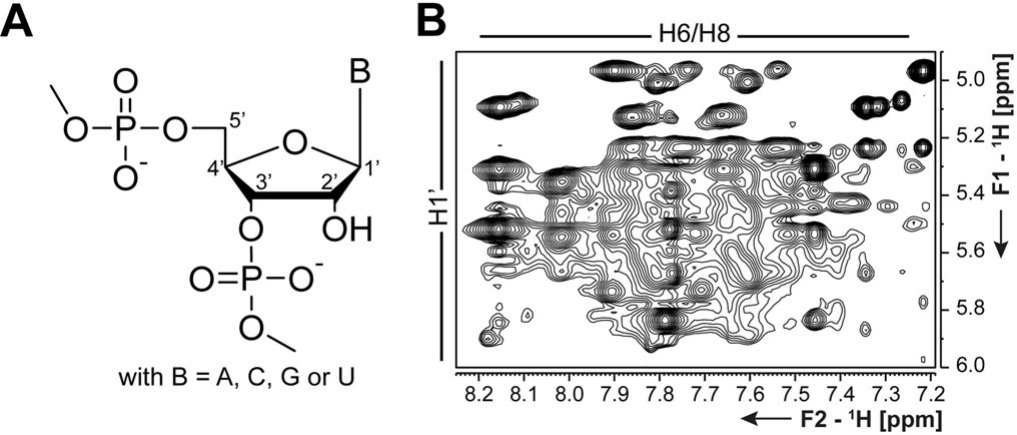
**A** RNA sugar phosphate backbone. **B** H1′‐H6/8‐region in a 2D H,H‐NOESY spectrum of a 41 nts long terminator hairpin of the 2′dG‐sensing riboswitch.^10^

One of the main drawbacks of NMR spectroscopy of RNA is the narrow chemical shift dispersion of the corresponding resonances due to the limited chemical diversity in building blocks (Figure 1 A). This limited dispersion leads to severe spectral overlap, which is particularly problematic in ^1^H‐spectra (Figure 1 B) and, therefore, puts a limit in molecular size of around 50 nts for NMR spectroscopy of RNA. However, when site‐selective labeling schemes are applied, the current limit is extended to 150–200 nts.^7^

Furthermore, compared to heteronuclei, the proton transverse relaxation rates are high and thus their line widths are large and increase fast with molecular size again impeding the characterization of larger RNA molecules. The nucleobases as heteroaromatic moieties possess few protons. Thus, only a few long‐range NOE contacts can be detected. Further, experiments that correlate NMR‐active nuclei by J‐transfer steps often require multiple and sometimes long magnetization transfer steps, in which sensitivity is lost due to relaxation processes. Examples for such experiments are the 3D TROSY‐relayed HCCH‐COSY experiment for the correlation of H2C2 and H8C8 in the adenine nucleobase^8^ as well as 3D HCCNH experiments for the correlation of the H6C6 (pyrimidines) or H8C8 (purines) with the imino protons in the nucleobases.^9^ Both of the experiments are crucial as they offer unique information for the sequential resonance assignment process. However, their long and multiple magnetization transfers are especially challenging for larger RNAs as relaxation is enhanced due to a larger rotational correlation time *τ*_c_.

The nucleobases are not only proton poor but the imino‐ and amino‐protons are often involved in different exchange processes. The imino proton is in fast exchange with the solvent water if not protected from exchange, mostly through hydrogen bonding or, in rare cases, other steric protection from exchange.^11^ This feature enables the fast determination of stable secondary structures, as only exchange protected imino protons can be detected. But it also leads to loss of information on dynamic regions of the RNA, as nucleobases in these regions are not involved in stable hydrogen‐bond networks and the imino proton resonances are broadened beyond detectability. Unfortunately, often the dynamic regions of an RNA are involved in functional processes including ligand‐ or protein‐binding.^5^, ^12^

Amino groups exhibit restricted rotation around the C−NH_2_ bond. The rates of rotation are similar to the chemical shift difference of the two amino protons, and the signals are thus broadened beyond detectability in this intermediate exchange regime.^13^

Taken together, both exchange processes of imino and amino protons severely hamper the collection of information on the orientation of the nucleobase, its potential interactions at the exchanging sites and their dynamics.

All four difficulties—1) resonance overlap, 2) low ^1^H‐density, 3) chemical exchange, and 4) relaxation—can be circumvented in heteronuclear‐detected NMR experiments. The disadvantage due to low‐γ detection can be minimized thanks to new cryogenic probes with inner NMR coils optimized for ^13^C‐, ^19^F‐, or ^15^N‐detection.^14^ Thus, despite their lower fundamental signal‐to‐noise ratio, heteronuclear‐detection schemes have become feasible. These heteronuclear‐detected experiments benefit from the larger chemical shift dispersion, coupled to sharper line widths of the heteronuclei. For example, ^13^C nuclei in RNA have chemical shifts from *δ*=65 to 170 ppm. If the chemical shifts of the heteronuclei are detected during direct acquisition, high resolution due to long FID sampling can be achieved without lengthening the experiments, as relaxation delays can be shortened.

The heteroaromatic nucleobases represent a cyclized chain of C−N fragments. This particular feature can be exploited for the direct magnetization transfer in NMR experiments in (multiple) INEPT steps without being dependent on ^1^H‐excitation or ^1^H‐detection. Information on quarternary carbon or nitrogen atoms then becomes feasible. Additionally, in heteronuclear‐detected experiments, the introduction of deuterium‐labeled nucleotides in large RNAs exploits their favorable relaxation properties in deuterium‐decoupled spectra^15^ but does not introduce the disadvantage of losing the observable nucleus. In addition, heteronuclei are not affected by solvent exchange.

Moving towards slower relaxing nuclei including ^13^C or especially ^15^N can bring potential advantages to extend molecular size limitation as line widths increase slower with molecular size when compared to ^1^H. On a technical side, further advantages of heteronuclear‐detection are their insensitivity to certain experimental conditions including pH value, temperature or salt concentration and the non‐necessity of water suppression.

The loss in sensitivity due to the lower gyromagnetic ratio is fundamental and remains, however, the major disadvantage in heteronuclear‐detected NMR experiments. Further, in uniformly isotope‐labeled samples, homonuclear J(C,C) couplings decrease the chemical shift resolution in ^13^C‐detected experiments. In particular, the sizeable homonuclear ^1^ *J*_CC_ couplings lead to splittings, as they are larger than the carbon line widths. However, decoupling schemes like IP/AP^16^ and S3E^17^ as well as selective homonuclear decoupling during acquisition are available.

## 2 Requirements: From NMR probes to sample preparation

Due to the reduced sensitivity of low‐γ‐detected NMR experiments, probes with cryogenically cooled detection coils and preamplifiers, so called cryogenic probes, are needed as they increase sensitivity about a factor of 3–4 when compared to room temperature probes.^14^ Probes that are optimized for ^13^C‐ or ^15^N‐detection are even better suited for recording heteronuclear‐detected experiments. As opposed to the so‐called inverse probes, which are used as standard probes in biomolecular NMR, they connect the channels of those heteronuclei to the inner coil of the probe enhancing the sensitivity for ^13^C‐ or ^15^N‐detection due to a larger filling factor. When working with fluorinated nucleotides, a two channel probe for ^19^F‐detection with simultaneous ^1^H‐decoupling is needed as *J*_HF_ scalar couplings tend to be large.

In biomacromolecular NMR of RNA, ^13^C‐ and/or ^15^N‐isotope labeled samples are indispensable.^18^ The simple and fast method of in vitro transcription using uniformly ^13^C‐ and/or ^15^N‐labeled rNTPs yields milligram quantities of RNA and is well established. For the characterization of larger RNAs, however, selective labeling schemes or the incorporation of modified nucleotides are often necessary.

Using mutants of the *T7* RNA polymerase during in vitro transcription allows incorporation of several modifications including rNTPs modified with fluorine or amino groups at the 2′‐position.^19^ While this method is independent of the RNA size, it is not specific as the modified nucleotide is incorporated uniformly in the RNA of interest. Position‐selective labeling of RNA (PLOR) overcomes this limitation and allows the automated enzymatic synthesis of position‐specific isotope‐labeled RNA by transcription.^20^ This method utilizes the possibility to pause and restart the RNA polymerase by omitting one nucleoside 5′‐triphosphate required for the transcription beyond a desired position. Although with this method milligram quantities of RNA with a desired isotope‐labeling scheme are obtained,^20^ the method is not commonly used because of its complexity and the lack of the commercial availability of the special apparatus.^21^

**Figure 2:**
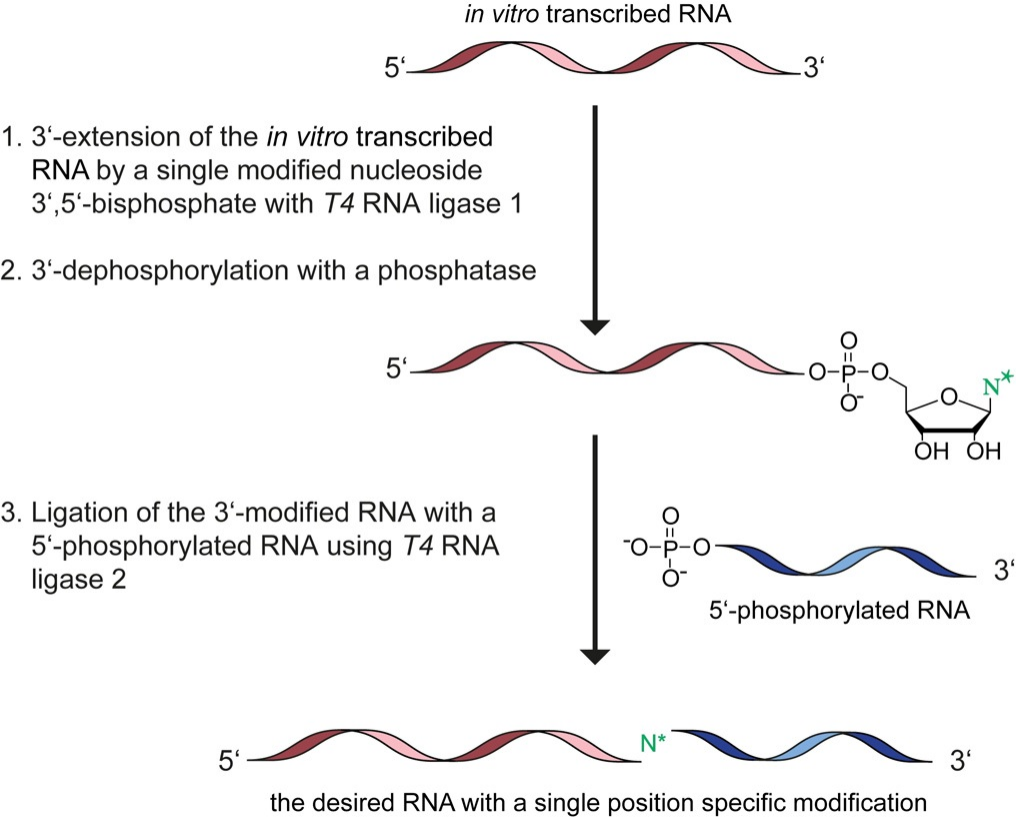
A three‐step chemo‐enzymatic procedure for the synthesis of position‐specific modified long RNAs. Transcribed RNA is extended at the 3′‐end with a modified nucleoside 3′, 5′‐bisphosphate using *T4* RNA ligase 1. After 3′‐dephosphorylation this modified RNA is elongated by *T4* RNA ligase 2 in presence of a 5′‐phosphorylated RNA.

Solid‐phase synthesis is one of the most commonly used methods for preparation of RNAs carrying a wide range of modifications.^22^ To overcome the NMR resolution problems position‐specific isotope‐labeled nucleoside phosphoramidites like 6–^13^C‐pyrimidine,^23^ 2′‐^13^C‐methoxy nucleoside,^24^ ^13^C_5′_‐sugar labeled nucleoside^25^ and ^15^N‐imino/amido nucleoside^26^ phosphoramidites are incorporated into RNA by chemical synthesis. However, this method is limited to RNAs of approximately 50 nucleotides^27^ for routine applications, taking the required amounts and purity into account. Alternatively, the genetic alphabet expansion technology allows the incorporation of unnatural base pairs that are compatible with the DNA polymerase and RNA polymerase allowing the amplification of modified nucleic acids by PCR and in vitro transcription.^28^ However, the unnatural nucleotide is incorporated into the DNA template by solid‐phase synthesis, which has again a size limitation. A reliable method for synthesis of site‐specific modified long RNAs are ligation‐based approaches using modified RNA fragments. Methyl transferases have been used to modify the 5′‐end of RNAs post transcriptionally.^29^ Also, the 3′‐end can be modified with nucleotidyl transferases that have the capability to incorporate modified nucleoside triphosphates^30^ or with *T4* RNA ligase 1 that is able to incorporate nucleoside 3′,5′‐bisphosphates with modifications at the sugar‐, phosphate‐, or base‐site.^31^ The latter allows the 3′‐extension of RNA by a single nucleotide, which in a further enzymatic step can be dephosphorylated at the 3′‐end using a commercially available phosphatase. Such RNAs carrying a hydroxyl group and a modified nucleotide at the 3′‐end undergo ligation with a 5′‐phosphorylated RNA in presence of *T4* RNA ligase 2 in an ATP‐dependent reaction. With this method, shown in Figure 2, RNAs up to a length of 390 nts containing a single position‐specific modification have been synthesized.^32^

## 3 ^13^C‐detection NMR spectroscopic experiments for RNA

The first reports utilizing carbon‐direct detection in protein NMR occured directly after the introduction of cryogenic probes.^33^ Applications to RNA started in 2007 with independent reports by Fiala et al.^34^ and Farés et al.^35^

### 3.1 Overcoming resonance overlap

**Figure 3:**
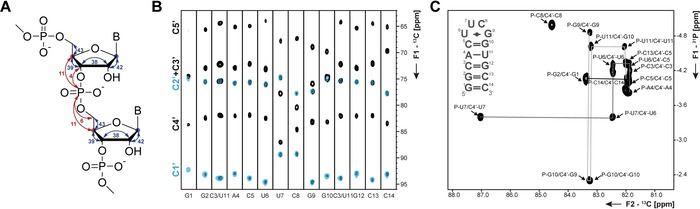
**A** Magnetization transfer pathway for the (H)CC‐TOCSY‐ (blue) and (H)CPC‐experiments (red). The size of ^*n*^ *J*(C,C) and ^*n*^ *J*(C,P) with *n*=1,2,3 utilized in the transfer pathways is indicated. Carbon nuclei at which magnetization is detected are marked with red and blue circles. **B** 2D planes of the 3D (H)CC‐TOCSY experiment with long (black) and short (blue) TOCSY mixing times. **C** 2D (H)CPC‐experiment for the C4′‐region of the 14nts RNA with UUCG tetraloop.

Due to limited chemical shift resolution, complete assignment of protons in particular for ribose protons is sparse in the BMRB database.^36^ The signals of ribose carbon atoms are much better resolved, so that carbon direct detection can contribute here towards increasing the number of assignments. 3D (H)CC‐TOCSY, (H)CPC‐ and (H)CPC‐CCH‐TOCSY‐experiments (see Figure 3 A for magnetization transfer pathway) exploit these favorable properties. The (H)CC‐TOCSY‐H1′C1′ experiment correlates all ribose carbon atoms with the C1′ chemical shift. By reducing the TOCSY mixing time one can further select for the C1′‐C2′ cross‐peaks and therefore differentiate between C2′ and C3′ chemical shifts (Figure 3 B). From the obtained carbon chemical shifts, the ribose conformation (C2′‐*endo* or C3′‐*endo*) can be determined.^37^

A sequential assignment from nucleotide to nucleotide can then be achieved in the (H)CPC‐experiment that correlates the C4′ resonance with the ^31^P chemical shifts in the 5′‐ and 3′‐site (Figure 3 C). Additionally, the phosphorus nuclei can be correlated with C1′ and C5′ nuclei by introducing a TOCSY‐ or COSY‐sequences.^38^ Compared to analogous ^1^H‐detected experiments,^39^ the ^13^C‐detected experiments have a slightly shorter magnetization transfer pathway. They are furthermore also applicable in partially deuterated samples and in particular C2′/C3′ assignment is facilitated. The (H)CC‐TOCSY experiment is furthermore not only applicable to RNA but to any kind of ribose‐containing molecules as shown by Fontana et al. for carbohydrates.^40^

### 3.2 Resonance assignment of nucleobase nuclei

^13^C direct‐detected experiments can also significantly contribute to complete resonance assignment of the nucleobases including their quarternary carbon or tertiary nitrogen atoms. There are two alternative approaches to assign these nuclei: a first suite of experiments exploits ^1^H‐excited and ^13^C‐detected experiments and the second suite exploits ^13^C‐excited and ‐detected experiments.

**Figure 4:**
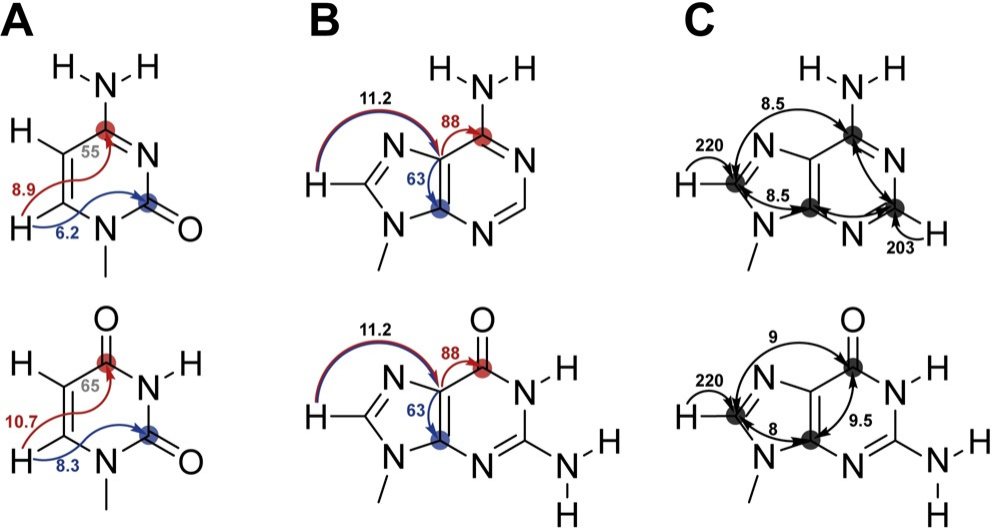
Magnetization transfer pathways for ^1^H‐excited and ^13^C‐detected 2D experiments; **A** Direct H‐C correlations in pyrimidines from H6 to C2 (blue) and C4 (red) via ^3^J(H,C) couplings, **B** H‐CC correlations in purines from H8 to C5 and C4 (blue) or C6 (red) and **C** HCC‐TOCSY in purines. The size of ^n^J(C,C), ^n^J(H,C) and ^n^J(N,H) with *n*=1,2,3 utilized in the transfer pathways is indicated. Coupling constants, used during IPAP decoupling schemes are depicted in gray. Carbon nuclei at which magnetization is detected are marked with a circle. Coupling constants have been taken from literature.^34^, ^41^

**Figure 5:**
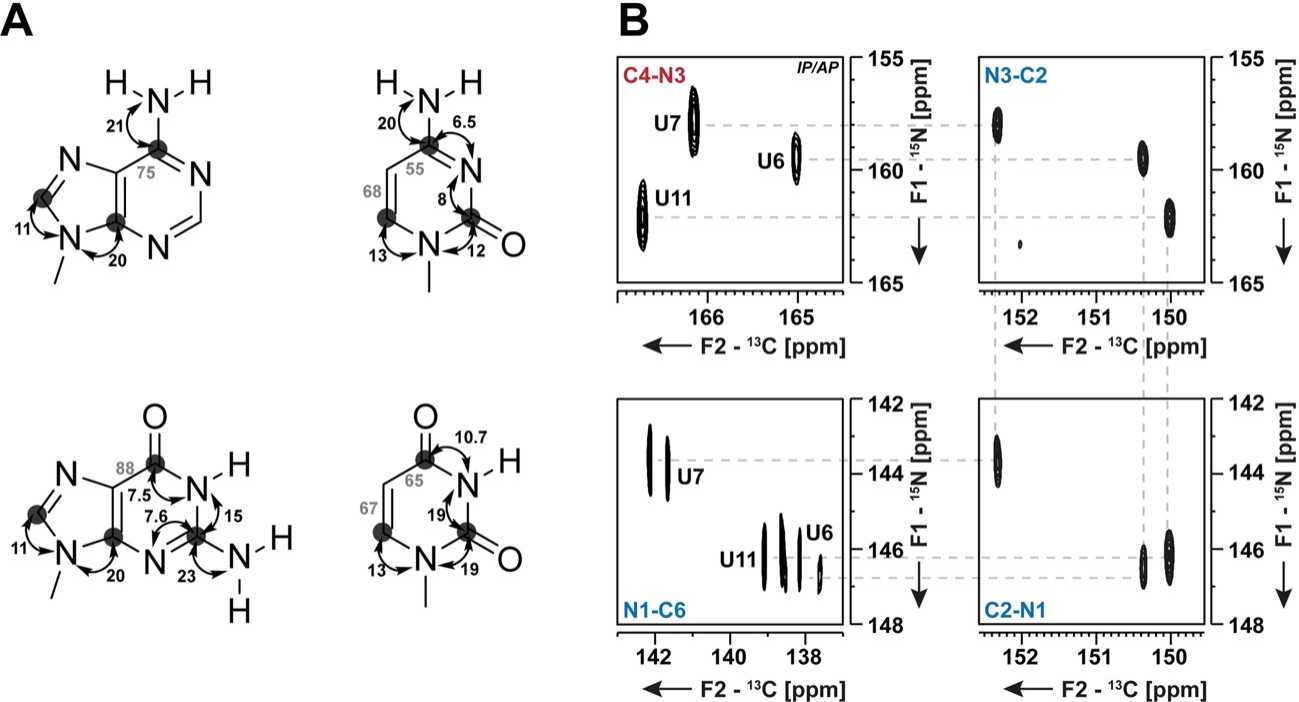
**A** Magnetization transfer pathway for the ^13^C‐excited and ^13^C‐detected 2D CN‐HSQC experiment. The size of ^1^ *J*(C,N) utilized in the transfers is indicated.^34^ Coupling constants, used during IPAP decoupling schemes are depicted in gray. Carbon nuclei at which magnetization is detected are marked with a circle. **B** Examples of 2D CN‐HSQC spectra for uridine residues in a 14 nts RNA with UUCG tetraloop. The walk through the nucleobase is indicated with a gray dashed line. The figure has been adapted from literature.^42^

In the first suite, the C2 and C4 in pyrimidines and the C2 (A), C4, C5, and C6 atoms in purines are assigned (Figure 4 A, B, C). The experiments give rise to C−H correlated spectra and are particularly valuable for larger RNAs as spectral overlap can be reduced through the carbon direct‐detection. In the second suite, not only quarternary carbon atoms but also tertiary nitrogen‐atoms are assigned using CN‐HSQC experiments (magnetization transfer Figure 5 A), provided the *J*(C,N)‐coupling constants are sufficiently large. For U, C, and G nucleobases, a near‐to‐complete resonance assignment can be achieved (Figure 5 B).^34^ However, the sequential walk through the C−N fragments of adenosines is hampered by the low ^1^J(C,N) couplings between C6N1, C2N1, C2N3, and C4N3 (Figure 5 A). The requirement of recording at least three different experiments with matched evolution times for the CN coherence transfer (29.4, 21.7, and 18.5 ms) to observe all nucleobase ^13^C and ^15^N atoms is the main disadvantage of this approach.^42^ Nevertheless, CN‐HSQCs have been successfully applied for the complete de novo assignment of a GTP‐binding aptamer with 39 nts.^43^

### 3.3 Determination of coupling constants

**Figure 6:**
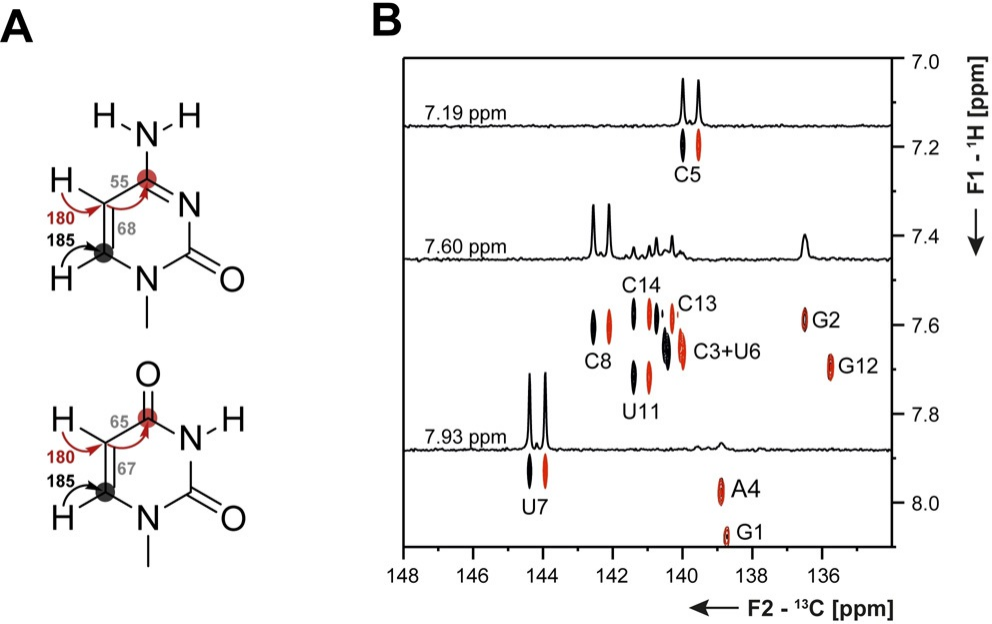
**A** Magnetization transfer pathways for ^1^H‐excited and ^13^C‐detected 2D experiments that correlate H6 to C6 (black) or H5 to C5 to C4 (red) to measure ^1^ *J*(C,C) coupling constants. The size of ^1^ *J*(C,H) and ^1^ *J*(C,C) utilized in the transfers is indicated. Coupling constants, which are determined in these experiments, are depicted in gray.^34^ Carbon nuclei at which magnetization is detected are marked with a circle. **B** 2D H6C6(C5) spectra for the measurement of the ^1^ *J*(C5,C6) coupling constant in pyrimidines. The figure was adapted from the literature.^34^

With the chemical shift assignments of ^13^C‐ and ^15^N‐nuclei in ^13^C‐direct‐detected NMR spectroscopic experiments, also *J*(C,C)‐ and *J*(C,N)‐coupling constants can be measured. ^1^ *J*(C,C) scalar couplings are determined, for example, in the H6C6C5‐experiment or in H5C5C4(C5)‐experiment (Figure 6 A).^34^ The *J*‐couplings are measured precisely through the deconvolution of the doublet splitting in the direct dimension (Figure 6 B). The obtained scalar couplings are in the range of ^1^ *J*(C5,C6)=67 or ^1^J(C4,C5)=55 Hz.^34^ Due to the high resolution in the direct dimension, the precision of the obtained values is high. Although this can be diminished by the inherent lower sensitivity of carbon detection. These experiments were applied in RDC studies of the TAR‐RNA.^44^

### 3.4 Detection of exchanging sites

**Figure 7:**
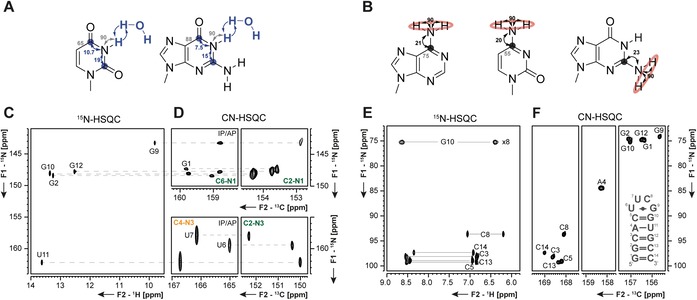
**A** Magnetization transfer pathways for the ^13^C‐excited and ^13^C‐detected 2D CN‐spin filter HSQC experiment^42^ and **B** the ^13^C‐excited and ^13^C‐detected 2D C(N)H‐HDQC experiment.^45^ The size of ^1^ *J*(C,N) and ^1^ *J*(N,H) utilized in the transfer pathways is indicated. Coupling constants, used during IPAP decoupling schemes are depicted in gray. Carbon nuclei at which magnetization is detected are marked with a circle. **C** ^15^N‐HSQC and **D** ^13^C‐detected CN‐HSQC spectra for the imino region of the 14 nts RNA with UUCG tetraloop. ^1^ *J*(C,N)‐coupling constants utilized for magnetization transfer were 17 (yellow) and 27 Hz (green). **E** ^15^N‐HSQC and **F** ^13^C‐detected CN‐HSQC spectra for the amino region of the 14 nts RNA with UUCG tetraloop (right).The figures have been adapted from Fürtig et al.^42^ and Schnieders et al.^45^

Typically, single‐stranded regions and nucleotides within dynamic secondary structure elements in RNA, such as long loops or bulges, cannot be observed in ^1^H‐detected experiments, because the imino proton reporter signals are broadened beyond detection by exchange with the hydrogen atoms from the solvent water (Figure 7 C, D). In order to overcome this blind spot in NMR of RNA, carbon‐detection experiments are utilized.

**Figure 8:**
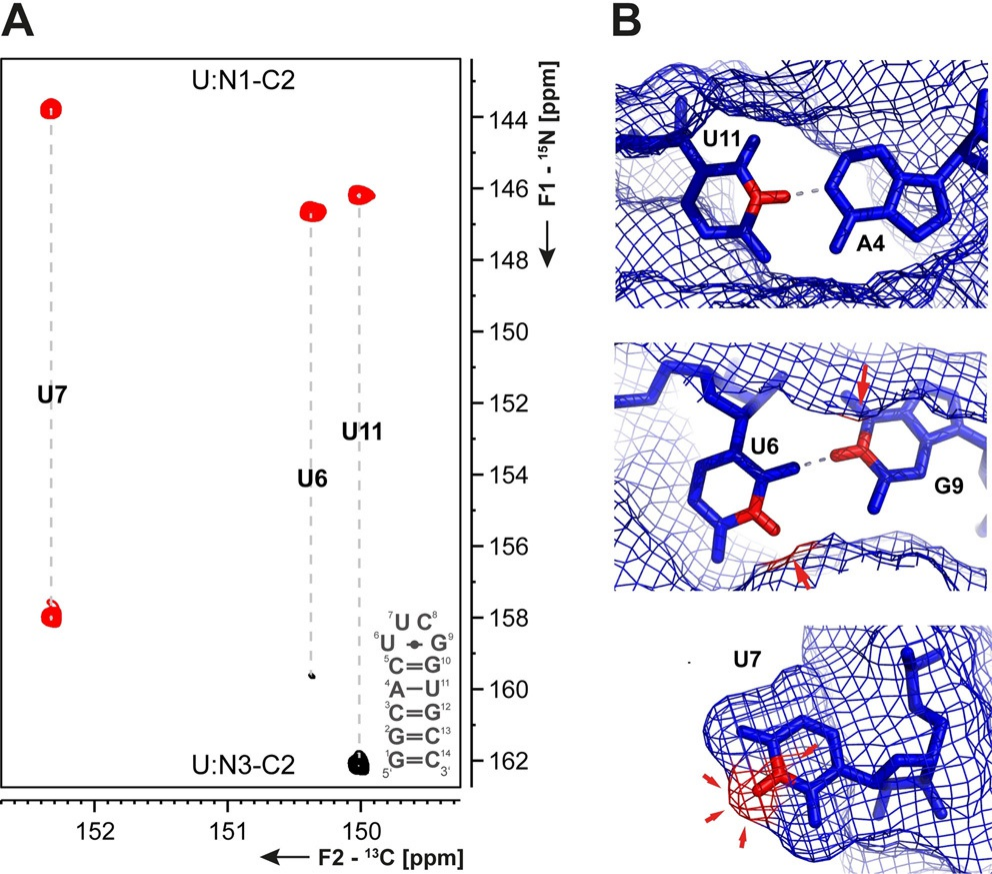
**A** 2D CN‐spin filter spectrum for the uridines of the 14nts hairpin RNA with UUCG tetraloop. **B** Structural context of the respective uridines (U11 WC base pair, H‐bonded imino proton; U6 sheared GU base pair, sterically shielded imino proton; U7 unpaired nucleotide, fully solvent exposed imino proton) are indicated.^48^ The figure has been adapted from the literature.^42^

The addition of a spin filter in the CN‐HSQC experiment (magnetization transfer Figure 7 A)^34^, ^46^ allows determination of the status of hydrogen bonding at the imino–nitrogen atom.^42^ The experiment makes use of the dependence of the scalar ^1^ *J*(N,H)‐coupling on the proton exchange rate. If the proton is in slow exchange with solvent water, the ^1^ *J*(N,H)‐coupling can evolve under an unscaled coupling of ≈90 Hz. If the proton is, however, in fast exchange with solvent water, the scalar coupling is decoupled through scalar relaxation of the second kind.^47^ Due to exchange, the spin state (α or β) of the imino proton is not maintained but changes with every H_2_O‐imino chemical exchange process. As a consequence, ^1^ *J*(N,H) is no longer observable. The spin filter has no effect for nucleotides with fast exchanging imino protons, whereas it inverts the ^15^N coherences for imino sites of nucleobases involved in stable interactions (Figure 8 A, B). As the signal intensity is thus modulated by the rate of proton exchange, the underlying exchange rates can easily be evaluated: the experiment allows quantitative determination of solvent exchange rates between *k*_ex_=10^0^ to 10^4^ s^−1^. The experiment relies on carbon direct‐detection. Therefore, imino exchange rates can also be measured even for samples dissolved in pure D_2_O, and kinetic isotope effects could potentially be determined.

**Figure 9:**
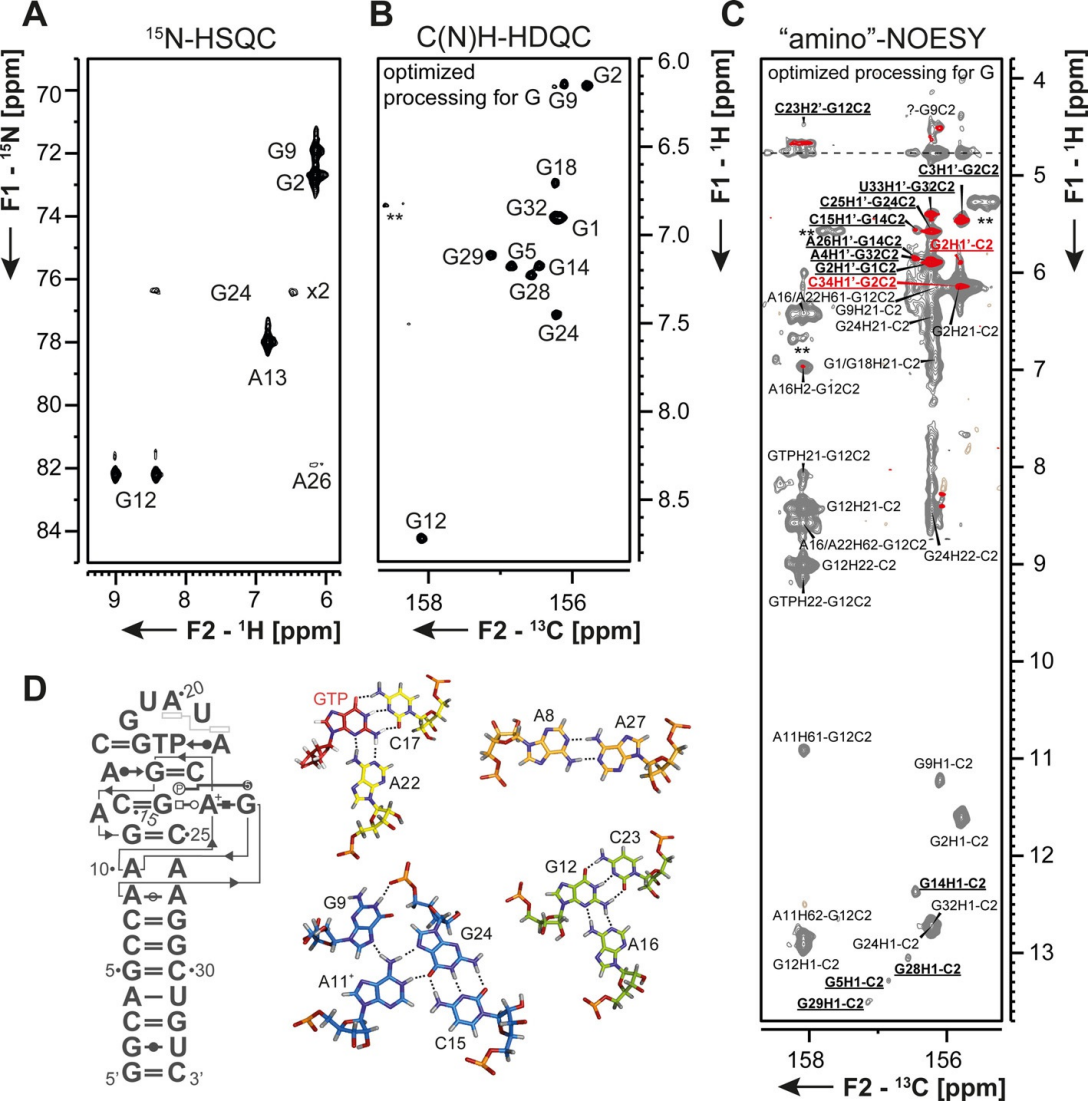
Comparison of **A** ^15^N‐HSQC, **B** C(N)H‐HDQC, and **C** “amino”‐NOESY spectra of the G‐amino region of the 34 nts long GTP class II aptamer^52^ RNA. **D** secondary structure and unusual tertiary nucleobase interactions of the RNA. The figure has been adapted from the literature.^45^ Experimental details can be taken from Schnieders et al.^45^

As opposed to imino groups, solvent exchange is negligible in amino groups.^49^ Here, a restricted rotation around the C−NH_2_ bond is often in intermediate exchange regime and renders the amino proton resonances undetectable (Figure 7 E, F). This is particularly prominent for adenosines and guanosines as seen in the ^15^N‐HSQC spectrum in Figure 9 A. Following an approach developed to detect nitrogen‐sites in the arginine side chains of proteins,^50^ new experiments to detect all NH_2_ groups in RNA have been developed.^45^ In these experiments, ^1^H‐double quantum (DQ) coherences are excited in the indirect dimension. This magnetization is transferred to the neighboring carbon atom, where it is detected (magnetization transfer Figure 7 B). Evolution of ^1^H‐DQ coherences is unaffected by chemical exchange and thus their line width is independent of bond rotation. With the ^13^C‐detected C(N)H‐HDQC experiment a C−H correlated spectrum is obtained, in which the ^1^H‐double quantum signals resonate at the mean proton chemical shift (Figure 9 B). This experiment enables the detection of a full set of sharp resonances for all amino groups independent of any kind of exchange.

Technical details require recording two independent experiments. During detection, the signal of the C6 in adenosines and the C4 in cytidines are doublets due to ^1^ *J*(C6,C5) values of 55 and ^1^J(C4,C5) of 75 Hz. Virtual decoupling schemes coined IPAP sequences remove these couplings that cannot else be decoupled for example, by homodecoupling. Thus, either two experiments are recorded with optimized values that match the respective *J*‐coupling or one experiment with an IPAP filter tuned to the averaged coupling of 65 Hz is recorded. The C(N)H‐HDQC represents a ^13^C‐detected experiment that allows detection of NMR signals that completely evade observation in proton‐based experiments.

Using ^13^C‐direct detection in tailored experiments allows also observation of NOE cross peaks from amino groups. The “amino”‐NOESY experiment (Figure 9 C) correlates protons and amino groups in NOE close proximity unobservable in conventional ^1^H‐detected NOESY experiment.^51^ The newly obtained NOE contacts often stem from H1′‐to‐amino‐group correlations.

In the refinement of RNA structure, they are of special value as they describe sequential and cross‐strand inter‐residual contacts. They significantly improve structure determination, in particular for dynamic RNAs.

### 3.5 Chemical‐shift‐to‐structure relations

Carbon‐direct‐detection experiments make chemical shift information accessible, which often is unavailable using proton‐detection. Chemical shifts are highly sensitive to the electronic environment of the respective nucleus and, therefore, they can potentially be used for a chemical‐shift‐to‐structure relation. In proteins this is already a standard method applied for the determination of secondary and even tertiary structure.^53^

Carbon‐chemical shifts of the ribose atoms are used for the determination of the ribose conformation in the RNA′s backbone. Based on an empirical calculation of the so‐called ‘canonical coordinates’ the ring pucker and the conformation of the exocyclic angle (O5′‐C5′‐C4′‐C3′: *γ*) can be extracted.^37^

Chemical shifts from ^13^C nuclei in the nucleobase do not depend on specific torsions but are sensitive to hydrogen bonding and stacking. With the first complete assignment of all carbon chemical shifts in nucleobases recorded by carbon‐detection experiments, a statistical analysis of the so far deposited chemical shifts in the BMRB database was undertaken in order to assign chemical shifts to structural context.^35^

**Figure 10:**
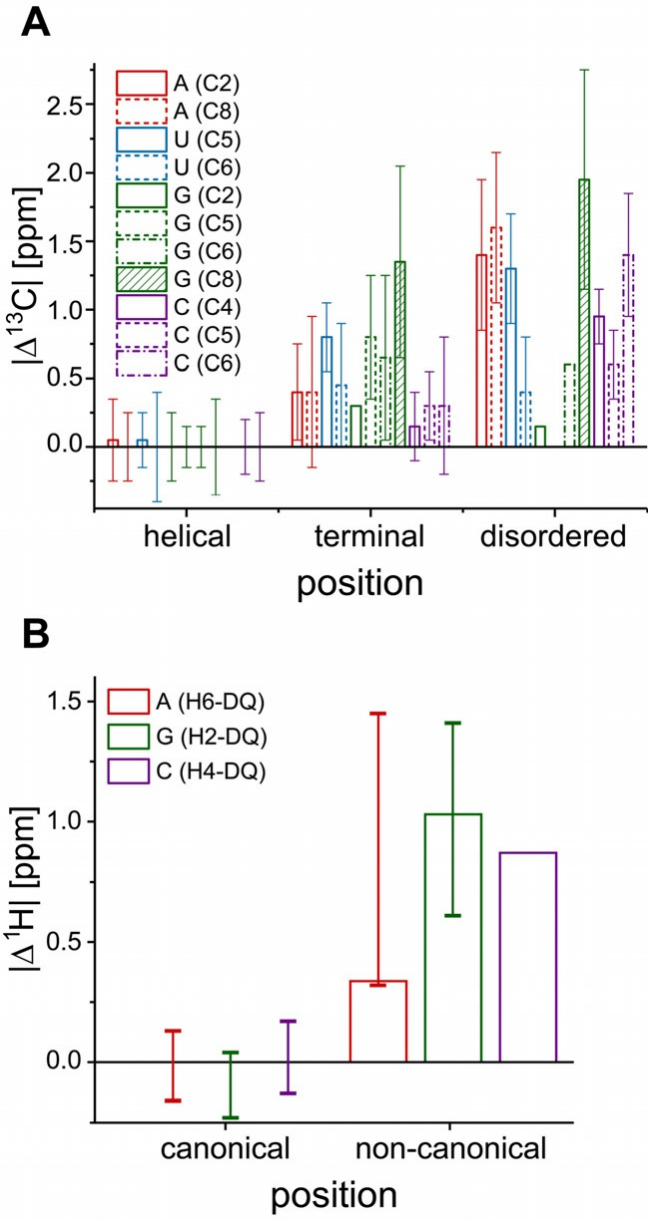
Absolute difference in the chemical shifts of **A** nucleobase ^13^C‐nuclei and **B** nucleobase ^1^H amino group nuclei in terminal and disordered nucleotides compared to nucleotides involved in helical interactions. The raw data was taken from Farés et al.^35^ and from Schnieders et al.^45^

The main effects that modulate the carbon chemical shift in the nucleobases are π‐stacking and hydrogen‐bonding interactions. Nucleotides can be classified into three different interaction type categories: helical (Watson–Crick base pairing and two site π‐stacking), terminal (Watson–Crick base pairing and one site π‐stacking), and disordered. All of the average chemical shifts of the atoms of the different nucleobases were referenced to the average chemical shift of the helical region and a clear trend can be observed for several carbon atoms in different structural elements (Figure 10 A).^35^ Also for the ^1^H‐DQ chemical shifts of the amino groups a chemical‐shift‐to‐structure relation was conducted.^45^ Similarly, as for carbon chemical shifts, a mean value was calculated for all nucleotides involved in Watson–Crick interactions. Therefore, nucleotides exhibiting a difference to this mean must be involved in a different interaction network. Analysis for five different RNAs allowed a clear discrimination between canonical and noncanonical base interactions (Figure 10 B).

## 4 ^15^N‐detection NMR experiments for RNA

^15^N‐direct‐detected multidimensional NMR experiments for RNA has only recently been introduced.^54^ Here, several ^15^N‐detected HN‐correlation experiments were applied to RNAs of increasing molecular size. This study was motivated by the development of ^15^N‐detection TROSY experiments for the analysis of proteins.^55^ In the field of proteins the experiments are particularly interesting for intrinsically disordered proteins (IDPs) as signals are usually very well dispersed in the ^15^N‐dimension, whereas the ^1^H‐dimension only covers less than *δ*≈2 ppm.

**Figure 11:**
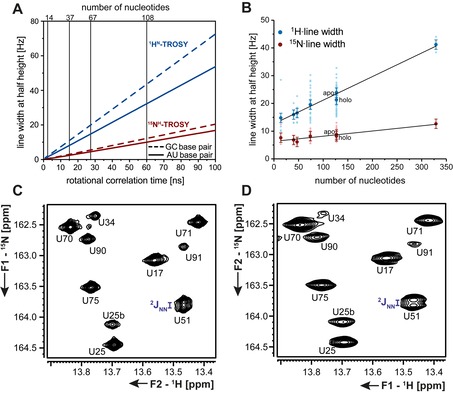
**A** Predicted line width at half height in dependence of the rotational correlation time for ^1^H^N^‐ and ^15^N^H^‐TROSY components. **B** Experimentally determined ^1^H and ^15^N‐line width at half height for RNAs of sizes between 14 and 329 nts. **C** ^1^H‐detected BEST TROSY spectrum and **D** ^15^N‐detected BEST TROSY experiment of the 127 nts long adenine‐sensing riboswitch,^56^ in which the ^2^ *J*(N,N) coupling across the hydrogen bond to the ligand can be resolved. The figure has been adapted from the literature.^54^

Similarly as for proteins, predictions for RNA show that line width of ^15^N‐resonances increases much slower with molecular size (rotational correlation time) than their proton counterparts. (Figure 11 A).

The ^15^N‐detection BEST–TROSY experiment was identified as the most sensitive ^15^N‐detection HN‐correlation experiment (Figure 11 C, D) and was thus applied to a set of RNAs ranging in size from 14 to 329 nts. The experimentally determined line width at half height confirmed the theoretical predictions concerning the trend in increasing molecular size and the differences in line widths for AU‐ and GC‐Watson–Crick base pairs (Figure 11 B). However, there was no improvement in sensitivity when compared to the ^1^H‐detection BEST–TROSY experiment. It might, however, be interesting to employ the ^15^N‐detection BEST‐TROSY experiment for the characterization of even larger RNAs due to the favorable relaxation properties.

## 5 Other nuclei

Besides the well‐established nuclei for heteronuclear‐detection schemes (^13^C, ^15^N), several experiments have been developed that detect magnetization of either non‐native nuclei (^19^F) or on rather exotic nuclei.

Although naturally occurring RNA nucleotides do not contain ^19^F‐nuclei, they can be introduced by means of chemical or biochemical synthesis at various positions (see the discussion above). It has previously been shown that fluorine modifications do not necessarily disturb the structure of the RNA, with the exception of fluorine labels at the 2′‐position.^57^ In the context of structured RNAs, the fluorine nuclei are then used as spy nuclei that show chemical shift perturbations over their wide chemical shift range in dependence of conformational changes.^58^ The great advantage of ^19^F experiments is that in biomolecular samples no background signals arise. However, ^19^F labelling comes also with two major disadvantages. The large chemical shift anisotropy (CSA) renders detection of ^19^F in large RNAs difficult. Further, the incorporated nucleotides are only ^19^F but not ^13^C labeled, so that only correlation experiments with proton‐nuclei can be recorded by exploiting the heteronuclear overhauser effect. Recently, these problems have been overcome by the introduction of nucleotides containing a pair of ^13^C–^19^F labels in the heteroaromatic nucleobases that allow recording of ^19^F–^13^C TROSY spectra.^59^

As a negatively charged biopolymer, interactions with cations are crucial for RNA/DNA folding and function.^60^ Those interactions can be characterized by NMR as relevant cations including Na^+^, Li^+^, and K^+^ are NMR‐active. NMR has extensively been conducted for DNA for which particular relaxation measurements have contributed towards understanding the nature of the interaction for Na^+^ and Li^+^ with double‐stranded DNA.^61^ In addition, G‐quadruplexes, for which cation binding is vital for formation, have been characterized using ^23^Na, ^39^ K, ^87^Rb, and ^205^Tl NMR spectroscopy.^62^ The latter is used as a substitute for K^+^ in the interaction with G‐quadruplexes in which different binding sites for the G‐quartets can be detected^63^ and even J(H,Tl) scalar couplings were measured.^64^

## 6 Conclusions and Outlook

The development of low‐γ‐detection schemes in NMR spectroscopy has been an active field over the last 15 years. Now, these possibilities are also exploited for NMR spectroscopy of RNA. Given the increasingly recognized biological relevance of RNA and the power of NMR spectroscopy to characterize its functional dynamics, the application of low‐γ‐detection schemes now allows forwarding NMR spectroscopy to larger RNA molecules. In order to reach this goal, development of advanced NMR methods runs hand‐in‐hand with improved methods in RNA sample preparation. The low‐γ‐detection schemes are further very compatible with solid‐state NMR experiments for RNA, as pioneered in the group of Carlomagno.^65^ The advantages of these novel direct detection methods for ^13^C‐ and ^15^N‐nuclei will become even stronger at higher magnetic fields that are now on the horizon.^66^

## Conflict of interest

The authors declare no conflict of interest.

## Biographical Information

*Robbin Schnieders*, *born in 1991, studied chemistry at the University of Frankfurt and finished studying in 2016 with her master's degree. Since then she has been working on her PhD in the group of Harald Schwalbe and is focused on the development of NMR spectroscopic methods for the characterization of RNAs*.

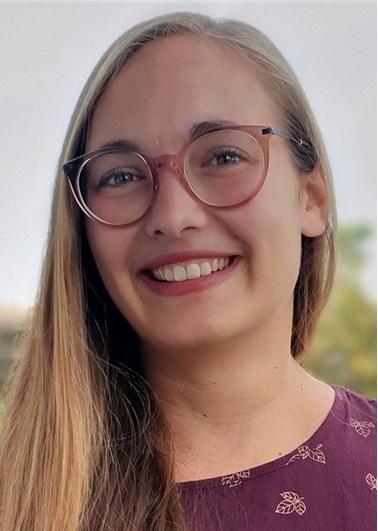

## Biographical Information

*Sara Keyhani, born in 1988, studied chemistry at the Goethe University in Frankfurt. She finished her PhD thesis in the group of Harald Schwalbe and holds, since then, a postdoctoral position in this group. Her research focusses on the development of new methods for the synthesis of RNAs and their non‐natural analogues for advanced biophysical studies*.

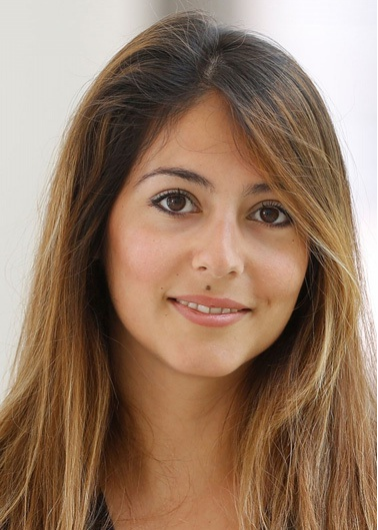

## Biographical Information

*Harald Schwalbe, born 1966, studied chemistry at the University of Frankfurt and obtained his PhD in 1993 with Christian Griesinger. He was postdoctoral fellow with Chris Dobson in Oxford from 1993–1995. After work on his habilitation till 1999, he became assistant professor at MIT from 1999–2001. In 2002, he accepted the offer to become full professor at Goethe University in Frankfurt. His research focussed on NMR spectroscopy of protein, RNA, and DNA molecules and their complexes. In particular, his group has made important contributions to NMR studies of RNA structure, dynamics, and folding*.

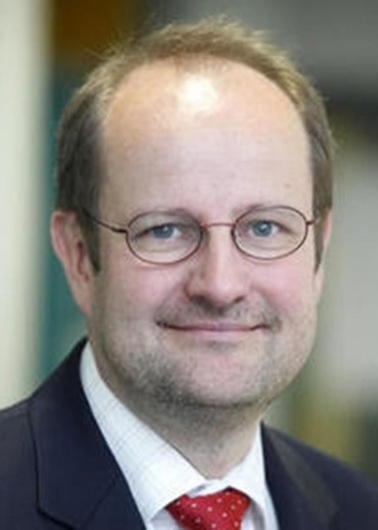

## Biographical Information

*Boris Fürtig, born 1978, studied biochemistry at the University of Frankfurt and obtained his PhD in 2007 with Harald Schwalbe. He was postdoctoral fellow with Renée Schroeder at the MFPL in Vienna and returned 2011 to Frankfurt University. His work focusses on the NMR‐based characterization of RNA dynamics*.